# Association Between Older Drivers’ Signs and Motor Vehicle Crashes in Japan

**DOI:** 10.2188/jea.JE20250386

**Published:** 2026-03-05

**Authors:** Masao Ichikawa, Haruhiko Inada

**Affiliations:** 1 Department of Global Public Health, Institute of Medicine, University of Tsukuba, Ibaraki, Japan; 2 Department of Public Health and Health Policy, Graduate School of Medicine, The University of Tokyo, Tokyo, Japan

**Keywords:** aging, automobile driving, policy, traffic collision

## Abstract

**Background:**

Encouraging older drivers to display the older drivers’ sign is one of the unique traffic safety policies for older drivers in Japan. The sign has been discussed in light of ageism, but the potential merit of displaying it has not been investigated.

**Methods:**

Using nationwide police-reported traffic crash data, we conducted a case-control study to examine whether displaying the sign is associated with reduced rear-end collision risk among drivers aged 70 years or older who were involved in car-to-car collisions from 2014 to 2023. The cases were those involved in rear-end collisions, the controls were those involved in angle collisions, and the exposure of interest was the sign display, which should be less often observed in the cases if the sign display is effective. The association was assessed with odds ratios adjusted for the time of crash and drivers’ sex and age group.

**Results:**

Among 74,433 cases and 13,885 controls, the proportion of those displaying older drivers’ signs was 38% and 39%, respectively. By the time of crash and drivers’ sex and age group, the proportion tended to be slightly higher in the cases than in the controls. In both cases and controls, the proportion was higher among women, in older age groups, and during daytime. The crude and adjusted odds ratios were 0.99 (95% confidence interval [CI], 0.95–1.02) and 1.08 (95% CI, 1.04–1.12), respectively.

**Conclusion:**

Displaying the older drivers’ sign was not associated with reduced rear-end collision risk. Alternative interventions are needed to ensure their traffic safety.

## INTRODUCTION

Ensuring traffic safety for older adults is an emerging public health challenge in many countries because of their aging populations. In Japan, a frontrunner of super-aged societies, unique licensing policies for older drivers have been introduced and revised over the decades.^1^^,^^2^ Today, older driver lessons and cognitive assessments are mandatory at license renewal for drivers aged 70 years or older and drivers aged 75 years or older, respectively. Drivers aged 75 years or older who violate certain rules, such as wrong-way driving and ignoring traffic signals, are required to take cognitive assessment right after the violation and a road test at the next license renewal. Additionally, older drivers are encouraged to display older drivers’ signs at the front and rear of cars to inform other road users of their presence. Installing a pedal misapplication prevention system in their cars and using cars equipped with an automatic emergency braking system are also recommended,^3^ while those who have safety concerns are encouraged to surrender their driving licenses.

**Figure 1.**
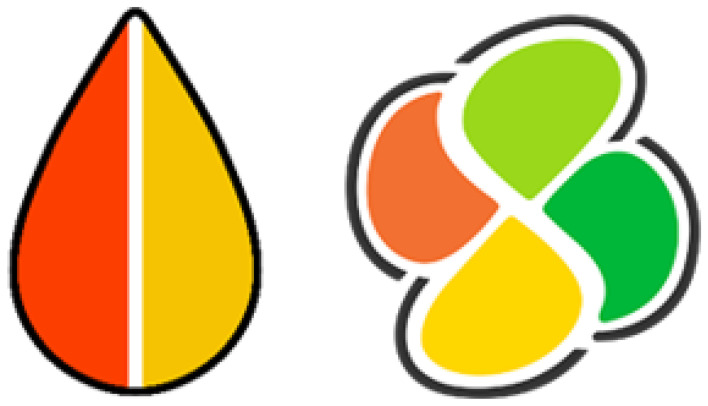
Older drivers’ signs. A leaf with two colors (orange and yellow) and a four-leaf clover with four colors (orange, yellow, green, and light green) were introduced in 1997 and 2011, respectively. Both signs can be used.

Older drivers’ signs were introduced for drivers aged 75 years or older in 1997 and drivers aged 70 years or older in 2001.^1^^,^^4^ Displaying the sign is optional now. Drivers around cars with the sign are obligated to keep a distance from the cars and not to cut in front of them. In 2008, displaying the sign was made mandatory but it was reverted the next year due to strong opposition from the public. The sign had been criticized because some people derogatorily reported that the sign looked like a mark of a fallen or withered leaf. In 2011, the design of the sign was changed from a leaf with two colors (orange and yellow) to a four-leaf clover with four colors (orange, yellow, green, and light green; Figure 1).

While the older drivers’ sign was discussed in light of ageism, the potential merit of displaying the sign for traffic safety has not received due attention. A previous study examining the prevalence of the sign display among older drivers involved in crashes between 2012 and 2017 reported that the older the drivers, the higher the prevalence, and the prevalence did not largely differ between at-fault and not-at-fault drivers.^4^ In 2017, the prevalence was around 40% among both at-fault and not-at-fault drivers, and it was around 30%, 50%, and 60% among drivers aged 70 to 74 years, 75 to 84 years, and 85 years or older, respectively. To our knowledge, car-to-car collision risk associated with displaying the sign has not been investigated. In the present study, we examined whether displaying the sign is associated with reduced rear-end collision risk.

## METHODS

### Study design

This is a proportional mortality study, a special type of case-control design. In this study, we used car crash data instead of mortality data. We hypothesized that displaying older drivers’ signs helps reduce rear-end collision risk because drivers following cars with the sign are supposed to keep a distance from the cars ahead and the sign is more visible at the rear than at the front of cars. Therefore, the cases are those involved in rear-end collisions, and the exposure of our interest is the sign display. We restricted the source population for the cases to a group of non-responsible drivers involved in car-to-car collisions. The advantage of this restriction is that non-responsible drivers, as they have no responsibility for the collisions, are considered to have an equal chance of being involved in any type of collisions (ie, a random sample), representing the driver population at risk of car-to-car collisions.^5^ We set two types of controls: one is those involved in angle collisions, assuming that these controls are unrelated to the exposure, and the other is those involved in collisions other than rear-end collisions for sensitivity analysis. We restricted the controls to those involved in angle collisions because they would be least likely influenced by the sign display, as explained below. If effective in reducing rear-end collision risk, the sign display should be observed less often among those involved in rear-end collisions (cases) than those involved in angle collisions and those involved in collisions other than rear-end collisions (controls). In proportional mortality studies, the proportional mortality ratio is commonly used to examine an association between the exposure and outcome. However, Miettinen and Wang (1981) and Rothman et al (2004) suggested a problem with the proportional mortality ratio and proposed to use an odds ratio as in a case-control study.^6^^,^^7^

### Data

We used nationwide police-reported traffic crash data from the Institute for Traffic Accident Research and Data Analysis. In Japan, police investigators collect crash information using uniform formats across the country, determining who was primarily responsible for the crash among those involved in the crash. In car-to-car collisions, drivers who were not primarily responsible for the crash could be contributory to the crash or not responsible for the crash at all. For analyses to be explained below, we defined “non-responsible drivers” as those not primarily responsible for the crash who had no record of traffic violations during that crash, assuming that their responsibility for the crash is minimal or none.

**Figure 2.**
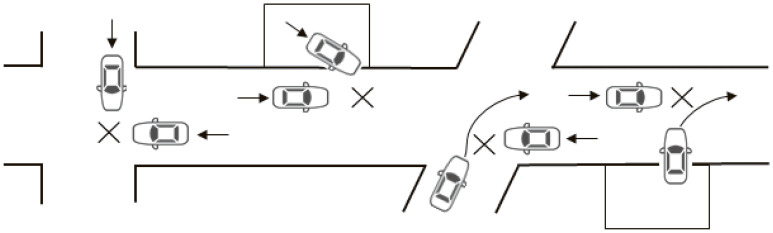
Examples of angle collisions

For this study, we obtained the number of car-to-car crashes of non-responsible drivers aged 70 years or older that occurred between 2014 and 2023, by their sex, age group (70–74, 75–79, or 80 years or older), the time of crash (daytime or nighttime), whether the sign was displayed at the time of the crash, and the crash type (head-on collisions, rear-end collisions while moving or while stopped, side-swipe collisions in the same directions or in opposite directions, angle collisions, angle collisions during left-turning, angle collisions during right-turning with oncoming cars going straight on the opposite lanes or in other situations, or others). Counterpart responsible drivers were of any age older than 18 years, the legal minimum age for driving a car in Japan. The crash type is the classifications used in the police-reported traffic crash data, and driving on the left is the norm in Japan. Angle collisions not during left- or right-turning are those that happen before or without turning, as shown in Figure 2. We assume that such angle collisions would be least likely influenced by the sign display because the sign may be unnoticeable in such abrupt collisions.

### Analysis

The effect of displaying the older drivers’ sign might vary by the time of crash and drivers’ sex and age because the sign might have smaller effects at night due to reduced visibility, and drivers’ sex and age might have potential unknown effects on the decision of displaying the sign and on the crash type due to their driving behaviors. Therefore, we compared the proportion of the sign display between those involved in rear-end collisions (cases) and those involved in angle collisions or those involved in collisions other than rear-end collisions (controls) by the time of crash (daytime or nighttime), drivers’ sex, and drivers’ age group (70–74 years, 75–79 years, or 80 years or older). The proportion in the cases would be smaller than that in the controls if the sign display had an effect. The strength of the association between the sign display and rear-end collision risk was assessed using logistic regression analyses with an odds ratios and 95% confidence intervals (CIs) adjusted for the time of crash, drivers’ sex, and drivers’ age group.

## RESULTS

During 10 years (from 2014 to 2023), 110,035 drivers aged 70 years or older were involved in car-to-car crashes as non-responsible drivers. Excluding 4,434 (4%) drivers for whom the display status of the older drivers’ sign was undetermined, 105,601 non-responsible drivers were subjected to the analysis. Of these drivers, 70% were male, 57%, 29%, and 14% were aged 70–74 years, 75–79 years, and 80 years or older, respectively; 86% were involved in crashes during daytime, and 38% displayed the sign. The crash type consists of rear-end collisions (70%), angle collisions (13%), head-on collisions (5%), angle collisions during right-turning with oncoming cars going straight on the opposite lanes or in other situations (3%), side-swipe collisions in the same directions or in opposite directions (2%), angle collisions during left-turning (1%), and others (6%). Table 1 shows the characteristics of 74,433 drivers involved in rear-end collisions (cases), 13,885 drivers involved in angle collisions (controls), and 31,168 drivers involved in collisions other than rear-end collisions (controls). The cases had a higher proportion of male drivers (71%) than the controls (62% and 65%) but other characteristics did not largely differ between the cases and controls, with the proportion of the sign display being 38% to 39%.

**Table 1.** Characteristics of non-responsible drivers involved in rear-end, angle, or other types of collisions from 2014 to 2023

	Total	Rear-end collisions (*N* = 74,433)		Angle collisions (*N* = 13,885)		Other collisions^a^ (*N* = 31,168)	
		
		*n*	%	*n*	%	*n*	%
Sex							
Male	73,417	53,056	71	8,562	62	20,361	65
Female	32,184	21,377	29	5,323	38	10,807	35

Age group							
70–74 years	60,672	43,640	59	7,458	54	17,032	55
75–79 years	30,305	21,167	28	4,063	29	9,138	29
≥80 years	14,624	9,626	13	2,364	17	4,998	16

Time of crash							
Daytime	90,919	63,446	85	12,381	89	27,473	88
Nighttime	14,682	10,987	15	1,504	11	3,695	12

Older drivers’ sign							
Display	40,327	28,486	38	5,364	39	11,841	38
No display	65,274	45,947	62	8,521	61	19,327	62

^a^Collisions (including angle collisions) other than rear-end collisions.

Table 2 shows the number and proportion of the sign display among the cases and controls by the time of the crash and drivers’ sex and age group. The proportion tended to be higher in the cases than the controls and differed between groups by 0 to 4 percentage points during daytime and 0 to 9 percentage points during nighttime, though caution should be exercised due to the small number of drivers in the older age groups during nighttime. In both cases and controls, the proportion was higher among women, in older age groups, and during daytime. The crude and adjusted odds ratios were 0.99 (95% CI, 0.95–1.02) and 1.08 (95% CI, 1.04–1.12), respectively, when the controls were those involved in angle collisions, and 1.01 (95% CI, 0.99–1.04) and 1.09 (95% CI, 1.06–1.12), respectively, when the controls were those involved in collisions other than rear-end collisions, suggesting that rear-end collision risk was slightly higher if the sign was displayed (Table 3).

**Table 2.** Number and proportion of non-responsible drivers displaying the older drivers’ sign among those involved in rear-end, angle, or other types of collisions, by the time of crash and their sex and age group, from 2014 to 2023

Sex	Age group	Crash type	Total	Daytime				Nighttime			
	
				Sub-total	Display	No display	% Display	Sub-total	Display	No display	% Display
Male	70–74 years	Rear-end	29,903	24,443	7,241	17,202	30	5,460	1,242	4,218	23
		Angle	4,255	3,601	1,010	2,591	28	654	112	542	17
		Others^a^	10,443	8,771	2,495	6,276	28	1,672	306	1,366	18

	75–79 years	Rear-end	15,414	13,268	5,834	7,434	44	2,146	738	1,408	34
		Angle	2,540	2,288	1,000	1,288	44	252	79	173	31
		Others^a^	6,085	5,412	2,309	3,103	43	673	218	455	32

	≥80 years	Rear-end	7,739	7,017	4,081	2,936	58	722	367	355	51
		Angle	1,767	1,651	975	676	59	116	56	60	48
		Others^a^	3,833	3,564	2,059	1,505	58	269	129	140	48

Female	70–74 years	Rear-end	13,737	11,882	4,405	7,477	37	1,855	628	1,227	34
		Angle	3,203	2,887	955	1,932	33	316	108	208	34
		Others^a^	6,589	5,860	1,965	3,895	34	729	233	496	32

	75–79 years	Rear-end	5,753	5,095	2,542	2,553	50	658	270	388	41
		Angle	1,523	1,397	656	741	47	126	46	80	37
		Others^a^	3,053	2,779	1,319	1,460	47	274	121	153	44

	≥80 years	Rear-end	1,887	1,741	1,056	685	61	146	82	64	56
		Angle	597	557	341	216	61	40	26	14	65
		Others^a^	1,165	1,087	647	440	60	78	40	38	51

^a^Collisions (including angle collisions) other than rear-end collisions.

**Table 3.** Association of displaying older drivers’ sign with rear-end collision risk, adjusted for non-responsible drivers’ sex and age group and the time of crash

	Rear-end vs angle collisions		Rear-end vs other collisions^a^	
	
	OR	95% CI	OR	95% CI
Older drivers’ sign				
Display	1.08	1.04–1.12	1.09	1.06–1.12
No display	Reference		Reference	

Sex				
Male	1.59	1.53–1.65	1.35	1.31–1.39
Female	Reference		Reference	

Age group				
70–74 years	1.53	1.45–1.62	1.39	1.34–1.45
75–79 years	1.33	1.26–1.41	1.24	1.19–1.29
≥80 years	Reference		Reference	

Time of crash				
Daytime	0.74	0.70–0.78	0.80	0.77–0.84
Nighttime	Reference		Reference	

CI, confidence interval; OR, odds ratio.

^a^Collisions (including angle collisions) other than rear-end collisions.

## DISCUSSION

Displaying the older drivers’ sign was not associated with reduced rear-end collision risk. The observed association was opposite of our hypothesized association. Though the sign may be helpful for other drivers to identify the presence of older drivers, it is uncertain whether the sign helped prevent older drivers from getting rear-ended by other drivers.

The negative association between the sign display and rear-end collision risk could be attributable to potential differences between older drivers displaying and not displaying the sign regarding their characteristics and driving exposures to certain road conditions that might affect collision risk. A previous study found that older drivers displaying the sign at the time of crashes had fewer traffic violations in the past than those not displaying.^4^ This finding implies that older drivers displaying the sign might have higher safety awareness and driving skills than those not displaying. However, we are unsure whether the effect of such characteristics on collision risk would appear differently for rear-end and other types of collisions. Moreover, it is uncertain whether driving exposures differed between those displaying and not displaying the sign.

Since displaying the older drivers’ sign would not affect their collision risk, it is up to them whether to display it, but our findings do not support making the sign display mandatory. To assist older drivers to drive safely, we should introduce potentially effective measures, such as using cars equipped with an automatic emergency braking (AEB) system^8^^–^^12^ and having passengers as co-pilots.^13^^–^^16^ In Japan, all cars manufactured since December 2025 (light trucks from September 2027) will be equipped with AEB.^3^ However, AEB cannot be retrofitted to the cars. Alternatively, a pedal misapplication prevention system that can be retrofitted is a viable option. Additionally, having passengers may be feasible for older drivers who have someone to go out within their family or neighborhood. We previously reported that the presence of passengers was associated with lower at-fault crash risk among older drivers even if they have cognitive decline.^17^ In the United States, some states can require medically-at-risk drivers to be accompanied by passengers as a strategy to reduce crash risk.^18^

We acknowledge several limitations. First, we could not ensure that the exposure is unrelated to the controls; in other words, the sign display may affect the risk of collisions other than rear-end collisions. Second, potential differences in the characteristics and driving exposures between those displaying and not displaying the sign could have biased the association between the sign display and rear-end collision risk. To ascertain the effect of the sign display, different study designs, such as regression discontinuity designs, may be applied, given the sign display once became mandatory. However, necessary data are not readily available for this study design. Finally, the potential effect of the sign on collisions for which older drivers had some responsibilities was not investigated in the present study. Such investigation is difficult because the collision situation, as well as traffic violations that make drivers responsible for collisions, may be too various to assess, while such data are not readily available.

In conclusion, display of the older drivers’ sign on vehicles was not associated with reduced rear-end collision risk. Alternative interventions are needed to ensure older drivers’ traffic safety.
